# Reactions with Wood Carbohydrates and Lignin of Citric Acid as a Bond Promoter of Wood Veneer Panels

**DOI:** 10.3390/polym10080833

**Published:** 2018-07-28

**Authors:** Claudio Del Menezzi, Siham Amirou, Antonio Pizzi, Xuedong Xi, Luc Delmotte

**Affiliations:** 1 Department of Forestry Engineering, Faculty of Technology, University of Brasilia, Brasília 70910-900, Brazil; cmenezzi@unb.br; 2 LERMAB, University of Lorraine, 27 rue Philippe Seguin, 88000 Epinal, France; siham.amirou@univ-lorraine.fr (S.A.); xuedong.xi@univ-lorraine.fr (X.X.); 3 IS2M, Institut de Science des Matériaux de Mulhouse, CNRS LRC 7228, 15, rue Jean Starcky, BP 2488, 68057 Mulhouse, France; luc.delmotte@uha.fr

**Keywords:** citric acid binder, adhesives, ^13^C NMR, MALDI ToF, esterification reactions, wood, wood veneers, LVL

## Abstract

The reaction of citric acid with wood veneers was studied by Cross Polarization Magic Angle Spinning Nuclear Magnetic Resonance (CP MAS ^13^C NMR) and matrix assisted laser desorption ionization time of flight (MALDI ToF) mass spectrometry. The analysis showed that reactions of citric acid occurred with both lignin and carbohydrate constituents of wood. The reactions occurring are esterifications between the carboxylic acid functions of citric acid and the numerous aromatic and aliphatic hydroxyl groups of the main wood constituents. Reaction of citric acid with glucose as a simple model compound of carbohydrates hydroxyl groups also yielded reactions leading to linear and branched oligomers by esterification. The result indicate that the reactions of esterification are accompanied in parallel by some internal rearrangements of lignin. The applied results on bonding wide flat wood surfaces such as veneers to obtain LVL panels yielded excellent strength results even if the conditions of pressing were more drastic than what is usual for this application. The applied bonding results have shown that citric acid has great potential to be used as a bio-binder for wood veneers.

## 1. Introduction

The development and interest in wood bonding systems based on renewable resources has increased considerably in the last few years. One of the most recent and interesting systems reported is the use of citric acid for promoting bonding of wood particleboards or other wood joints. After the original research group discovering the effect of citric acid [1], several authors have shown the potential of this natural product to bind particleboards using several types of biomass [2,3,4]. Citric acid has also been found to provide improved dimensional stability for particleboards and to markedly enhance the water resistance of wood-welded joints [5]. Citric acid acts as a bond promoter at different levels of wood joints, the real adhesive being polymeric wood constituents mobilized by its presence and temperature.

Nevertheless, to bond the flat surfaces of plywood or of laminated veneer lumber (LVL) is a rather different application situation. No research dealing with the utilization of citric acid for bonding wood veneers for LVL and for plywood has been carried out as yet. Thus, the work presented here deals in part with the application of citric acid as a bond promoter for laminated veneer lumber and plywood.

While a few articles in the literature [1,2,5] have addressed the reasons why citric acid should have such a binding effect, the chemical reasons underlying its mechanism of action are not really clear. After analysis, some research groups have advanced the hypothesis of citric acid esterifying hydroxyl groups in the wood constituents, and thus forming cross-links across wood constituents, and this included across the bonding interface [1,2]. According to the studies of Umemura et al. [1] and Kusumah et al. [2], the ester linkages between the carboxyl groups derived from citric acid and the hydroxyl groups of wood components bring adhesiveness thus causing good physical properties. Other groups using citric acid for a different application and under very different reaction conditions [5] have indicated that the changes observed in CP-MAS ^13^C NMR spectra of welded spruce pretreated with citric acid rather show that citric acid has also a strong catalytic effect. The improved bonding and water repellency was mainly ascribed in this case to the rearrangements and recombination of lignin and other wood constituents. Both these explanations may not present the complete picture nor be completely satisfactory. Thus, as the development of such a fundamentally easy-to-use and inexpensive bio-binding system is of particular interest, the work presented here is also aimed at further studying and clarifying what does occur at the chemical level when citric acid acts on wood at the curing temperatures generally used for wood panels.

## 2. Materials and Methods

For chemical analysis two sets of specimens were prepared. For one set of samples, poplar veneers were treated with 20% citric acid based on the veneers’ weight and then dried in the oven at 60 °C for 6 h. The second set of samples was prepared from the pressed boards after testing them. The bonded joints were reduced to powder by sanding and limiting it to a very small depth to limit the predominance of the unaffected wood in the spectra.

### 2.1. Cross Polarisation-Magic Angle Spinning Nuclear Magnetic Resonance (CP-MAS ^13^C NMR) Spectra

The surface of the citric acid bonded joints obtained was analyzed by solid state CP MAS ^13^C NMR. The equipment was used to analyze the surface of the open joints after mechanical testing. The bonded joints were reduced to powder by sanding and limiting it to a very small depth to limit the predominance of the unaffected wood in the spectra. The spectra were obtained on a Brüker Avance 400 MHz spectrometer (Brüker, Billerica, MA, USA) at a carbon resonance frequency of 100.6 MHz. The impulse duration at 90° was for 4 microseconds. The rotor was spun at 12 kHz on a double-bearing 4 mm Brüker probe. The cross polarization contact time was 1 ms and a ramp 100% was used with a continuous wave decoupling. The number of scans was from 4000 to 10,000. The spectra of the untreated sample and the sample treated with 40% of citric acid for 1 h at 103 °C and of the sample obtained from the pressing of panels at 180 °C are reported.

### 2.2. Matrix Assisted Laser Desorption Ionization Time of Flight (MALDI-TOF) Mass Spectrometry Analysis

The fine powder samples were treated with a NaCl solution (1.5 µL of a 0.1 M), and a methanol/water mixture (1:1) was added to increase ion formation, and placed on the MALDI target and dried. The samples and the matrix were then mixed in equal amounts, and 1.5 µL of the resulting slurry was placed on the MALDI target. A matrix of 2,5-dihydroxy benzoic acid was used. Red phosphorous (500–3000 Da) was used as reference for spectrum calibration. Finally, after evaporation of the solvent, the MALDI target was introduced into the spectrometer. To more clearly see the reaction of carbohydrates with citric acid, glucose was also used as a simple model compound of carbohydrates, and it reacted with citric acid, the reaction product being analyzed by MALDI ToF. Glucose reacted with citric acid in the proportion of glucose: citric acid 1.5:1 to which were added 0.5 a part of water to dissolve the glucose, and it reacted at 100 °C for 1 h. After reaction, a sample of the liquid was placed on the MALDI sample carrier and dried at 40 °C for 2 h before being analyzed.

The spectra were recorded on a KRATOS AXIMA Performance mass spectrometer from Shimadzu Biotech (Kratos Analytical Shimadzu Europe Ltd., Manchester, UK). The irradiation source was a pulsed nitrogen laser with a wavelength of 337 nm. The length of one laser pulse was 3 ns. Measurements were carried out using the following conditions: polarity-positive, flight path-linear, 20 kV acceleration voltages, and 100–150 pulses per spectrum. The delayed extraction technique was used applying delay times of 200–800 ns. The software MALDI-MS was used for the data treatment. The oligomers can appear in the spectra either corresponding to their molecular weight or to their molecular weight +23 Da of the Na^+^ ion derived from the NaCl used as enhancer. The spectra precision is of ±1 Da.

### 2.3. Wood Veneer Board Making and Testing

Initially 10 veneers (MC = 10.3%; ρ = 449 kg/m^3^) measuring 400 mm × 400 mm × 2–3 mm from poplar wood (*Populus* sp.) were selected, weighted, and measured. A citric acid/water solution (50:50) was prepared and sprayed on the one surface of the veneers until a load of solution of about 134 g/m^2^ single glue line was obtained, and then they were oven dried at 60 °C for 6 h. The assembling of the LVL billet was done so that citric acid treated veneer surface faced the opposite untreated surface veneer. Afterwards, the 5-layer LVL billet (400 mm × 20 mm × 10.5 mm) was hot-pressed using a 4-step pressing schedule: 180 °C, 1.5 MPa for 5 min; 3.0 MPa for 10 min; 1.5 MPa for 2.30 min, and 0.5 MPa for 2.30 min. This schedule was applied aiming at the consolidation and densification of the board at same operation and it was performed using a Joos Lap 150 (Pfalzgrafenweiler, Germany) laboratory-press. An INSTRON 4467 universal testing machine was used to assess bending properties (modulus of rupture, *f*_m_, and modulus of elasticity, *E*_M_) and compression parallel to the grain strength (*f*_c,0_) according EN408 + A1 [6]. Because of the dimensions of the board the (*f*_c,0_) width was 25 mm, but the length was six times the board thickness. The bonding quality was evaluated by assessing the glue-line shear strength (*f*_gv,0_) at dry conditions according to EN314-1 [7] for bond quality according to EN314-2 [8]. Thickness swelling and water absorption (TS/WA) tests were performed according to ASTM D1037 [9] procedures, but the dimension of the samples was changed: 25 mm × 25 mm × thickness. Permanent thickness swelling (PTS) and equilibrium moisture content (EMC) were determined according to Del Menezzi et al. [10]. From the LVL board, a small piece of the glued joint (18 mm × 6 mm × 2 mm was cut and it was covered with gold before scanning electron microscopy (SEM) analysis. The analysis was performed with a Hitachi Tabletop Microscope TM3000 (Hitachi High Technologies, Velizy, France) under vacuum, at a working distance of 6.4 mm and 15 kV accelerating voltage. The density profiles of the board were determined by using a Grecon Da-X (Greten, Germany) density profiler.

## 3. Results and Discussion

**Figure 1:**
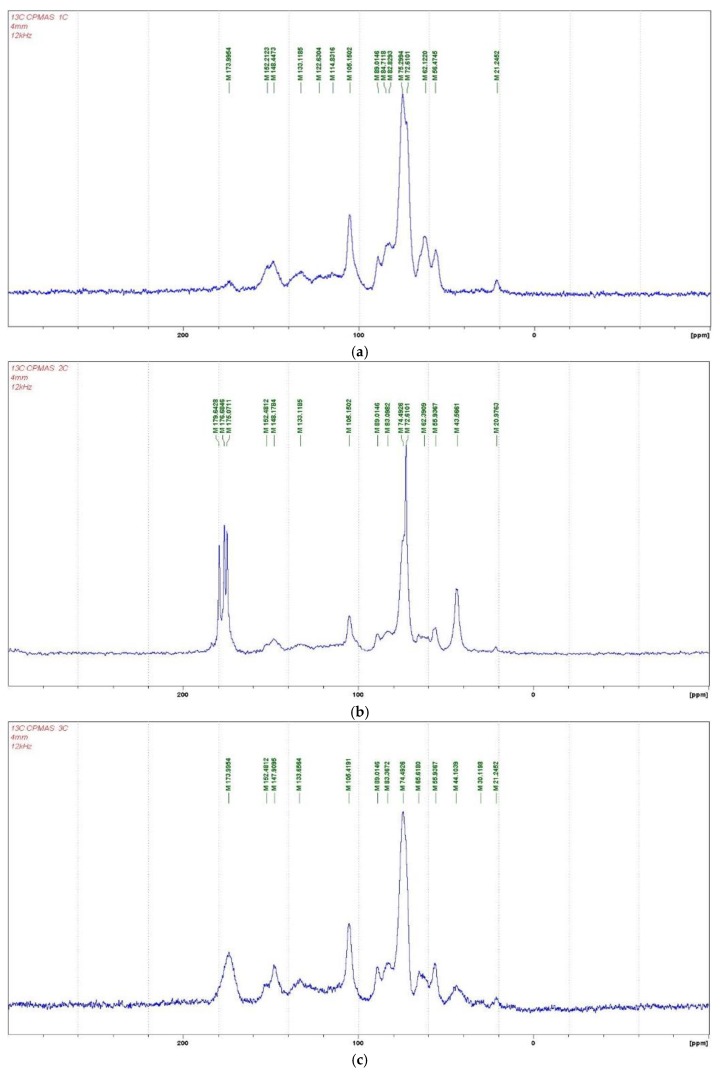
CP-MAS ^13^C NMR spectrum of (**a**) wood alone; (**b**) wood treated with citric acid at 60 °C for 6 h; and (**c**) wood taken from cured glue line of veneers panels treated with citric acid and pressed at 180 °C.

**Figure 2:**
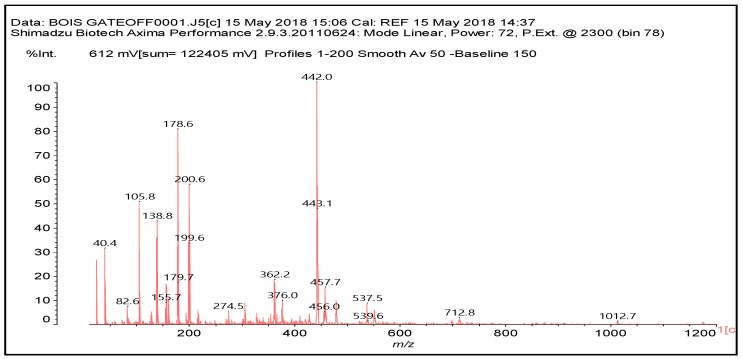
MALDI ToF spectrum of wood alone. 20–1200 Da range.

**Figure 3:**
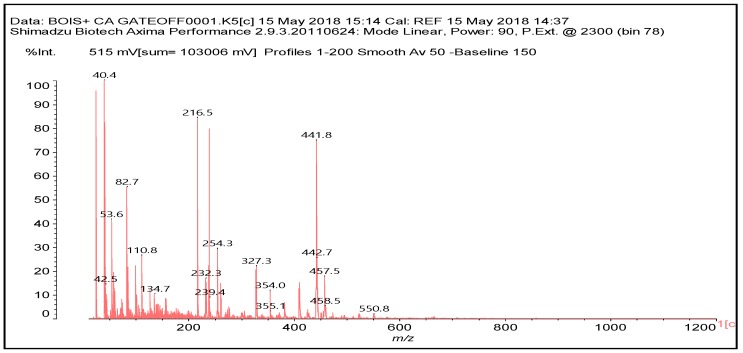
MALDI ToF spectrum of wood and citric acid at 60 °C. 20–1200 Da range.

The CP MAS ^13^C NMR spectra (Figure 1a–c) of the reaction products obtained by the reaction of wood with citric acid at 60 °C, as well as for wood particles reacted by hot pressing at a temperature of 180 °C, gave an initial indication of the reactions occurring between some wood constituents and citric acid. The more evident signs of reaction between wood and citric acid can be observed in the wood and citric acid hot-pressed specimens. In these, one of the main indications of reaction is the small band at 30 ppm. If citric acid unreacted has the following ppm shifts (structures I, and II if protonated), as reaction occurs, the shifts change indicating a reaction between the phenolic hydroxyl groups of lignin with citric acid. From the shifts above and below (structures III–VI) this is shown by the small band at 30 ppm in the 180 °C pressed case spectra. All the other bands are characteristics of lignin and other groups of citric acid. The wide band between 180 and 170 ppm in the 180 °C pressed wood/citric acid spectrum (Figure 3) and the multitudes of different carboxyl bands in the 60 °C case spectrum (Figure 2) are an indication of different environments in which the carboxyls of citric acid and of the citric acid aromatic esters find themselves. Thus, in the 60 °C reaction case (Figure 2), the 175, 177, and 179–180 ppm bands were respectively the bands of the carboxyl of the aromatic ester or of the –COO–, of the unreacted –COOH of citric acid and of the protonated –COOH^2+^ of citric acid. The decrease of the bands at 122 and 114 ppm of the aromatic nuclei of lignin once treated with citric acid can indicate either a substitution of the ArC–H on the aromatic ring with an ArC–C; however, there is no other band in the spectra supporting this idea. It must be equally noted that the substitution can also be on alpha and beta carbons of the aliphatic chain of lignin.


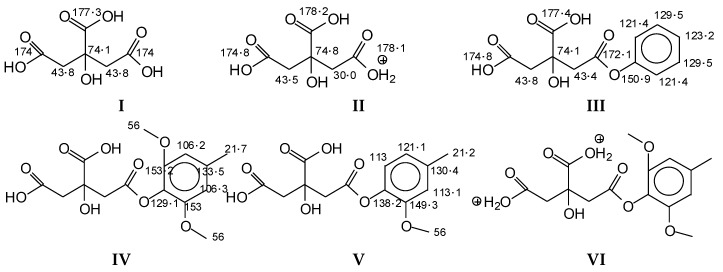


Thus, all that can be said from the CP MAS ^13^C NMR is that reaction occurs between some of the free phenolic hydroxyl groups of guayacyl type lignin units and citric acid. There are indications that the hydroxyl group on the aliphatic chains of lignin units are also able to react with citric acid, as it would be expected in an esterification, as these have a predominantly alcohol like behavior. This is the same for the hydroxyl groups of the wood carbohydrates. In Figure 3, the peak at 65 ppm was also markedly decreased by the acid citric treatment. This peak belongs to the –CH_2_OH of the aliphatic side chains of lignin as well as to the C6 of the –CH_2_OH of hexose carbohydrates, thus cellulose and the two main hemicellulose types. It means that –CH_2_OH groups from either lignin and/or the carbohydrates or from both have reacted in some way. This is an indication that esterification may well have occurred. Part of the decrease in CH_2_OH groups may also be due to the cleavage of this group from lignin or carbohydrates to form methanol, which should transmit at 46–49 ppm, this being possibly the shift observed at 44–46 ppm. This has been reported previously as a possible indication of internal rearrangements of lignin [5].

Figure 3 shows also that the peak at 174 ppm is wide corresponding to more than one carboxyl group, namely that of citric acid at 166–167 ppm, and of its esters issued of its reaction with different hydroxyl groups of wood lignin and carbohydrates.

**Figure 4:**
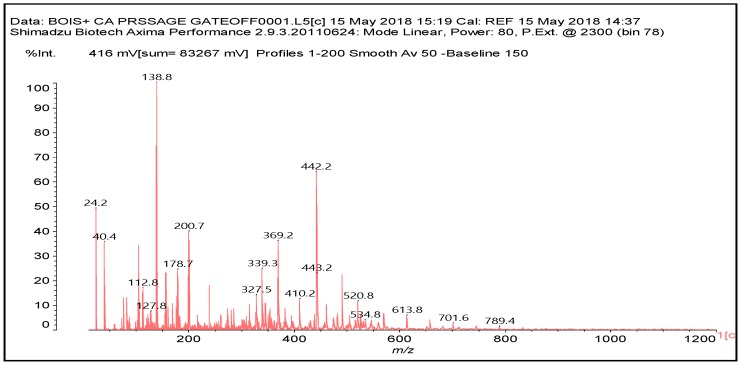
MALDI ToF spectrum of the wood and citric acid hot-pressed at 180 °C. 20–1200 Da range.

Confirmation of the insights gained by NMR analysis and further indications of the reactions occurring can be obtained by the MALDI ToF analysis of some of the same specimens above. The spectra in Figure 2, Figure 3 and Figure 4 shows that the more clear indications of reaction are in the 180 °C hot-pressed wood and citric acid specimens. Several peaks confirming co-reaction of citric acid with lignin were observed such as the peak at 613.8 Da (structure VII, calculated 591 + 23 = 614 Da)


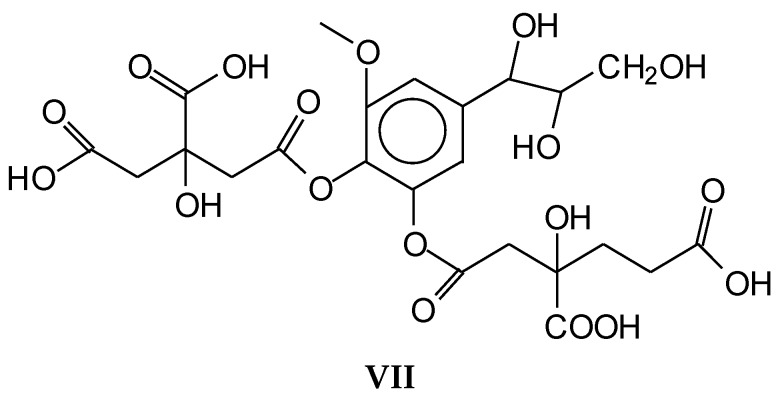


And also the peak at 789.4 Da (structure VIII, calculated 766.2 + 23 = 789.2 Da)


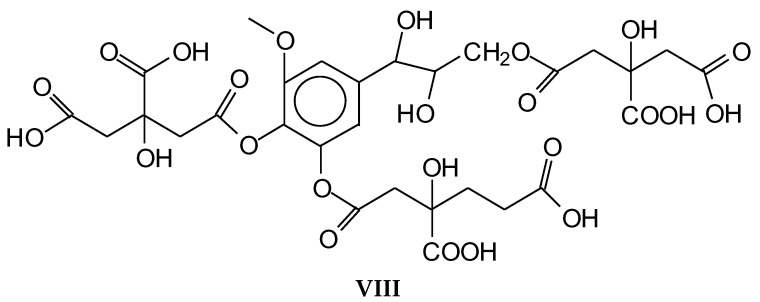


Structure VIII is proof that also the alcohol side chain –OHs of a lignin unit can react with citric acid.

There are other peaks that could belong to the reaction of citric acid with guajacyl and syringyl lignins, but unfortunately they are also present in the wood spectrum alone. These are the cases of the peak at 442 Da (a guajacyl lignin unit reacted with one citric acid) and the peak at 457 Da (a syringyl lignin unit reacted with one citric acid).

Furthermore, there is a peak at 550.8 Da (calculated = 528 + 23 Na^+^ = 551 Da) in the 60 °C wood-treated case spectrum (Figure 3) of the reaction that belongs to a mixed citric acid-glucose-citric acid oligomer of the type (structure IX)


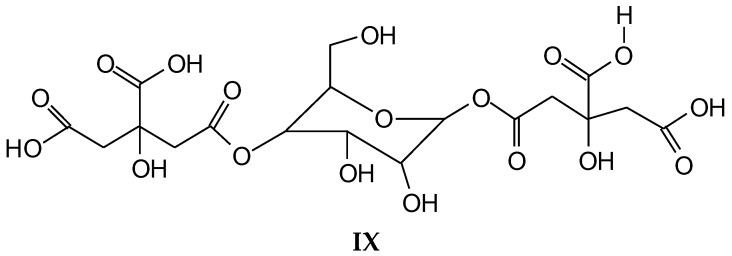


Again this indicates that carbohydrates and polymeric carbohydrate in wood also react with citric acid.

In the MALDI spectrum of the 180 °C hot-pressed specimens there are two series of peaks of interest. One is the 657–833–1009–1185 Da where the peaks are separated by repeating intervals of 176 Da, this corresponding to a citric acid residue, implying a basic compound formed at 657 to which is added a long linear chain of citric acid residues or much more probably a piece of hemicellulose at 657 Da the –OHs of which are esterified by three citric acids.

There is a concurrent series of peaks at 657–818–981–1143 Da where the peaks are separated by a regular repeating interval of 162 Da, this corresponding to a glucose chain.

The question is now: what does the 657 Da peak represent? This seems to correspond to a three glucoses chain to which is attached just one citric acid, thus glucose-glucose-glucose-citric acid, calculated 660 Da without Na+ that if bi-deprotonated would give 658 Da. Thus, it is on this compound that two different series are formed. One with a three glucoses chain on which four citric acids are attached each to a different –OH, thus the series 657–833–1009–1185 Da. The second series of compounds which also occurs is formed by chains of respectively 3, 4, 5, and 6 glucoses to each of which there is only one single citric acid residue attached.

**Figure 5:**
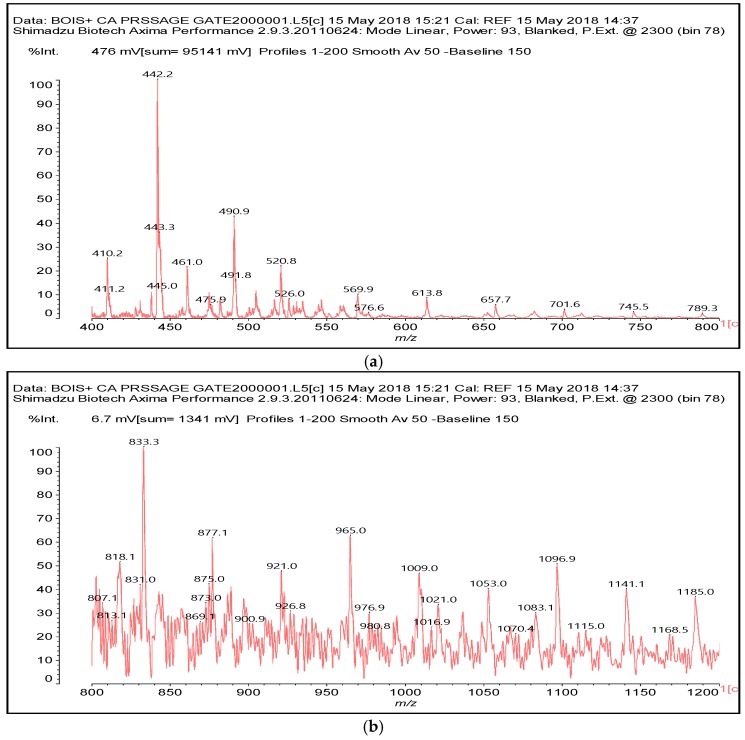
MALDI ToF spectrum of the reaction of glucose with citric acid. (**a**) 400–800 Da range; (**b**) 800–1200 Da range.

While indication of reaction with the aromatic and aliphatic hydroxyl groups of lignin has appeared from the NMR analysis, the MALDI ToF analysis confirms the reaction of carbohydrates and carbohydrate oligomers with citric acid. To further confirm this point, glucose was used as a simple model compound of carbohydrates and reacted with citric acid. The resulting reaction mixture was analyzed by MALDI-ToF showing clearly the formation of a number of glucose-citric acid oligomers. Thus, the following relevant peaks could be identified in Figure 5a,b indicating that the reaction of esterification does occur with ease.

The following peaks, attesting that the reaction occurs, can be seen:

- 352 Da = glucose-citric acid
- 518 Da = glucose-citric acid-glucose (no Na^+^)
- 527 Da = citric acid-glucose-citric acid (no Na^+^)
- 539 Da = glucose-citric acid-glucose (with Na^+^), structure X
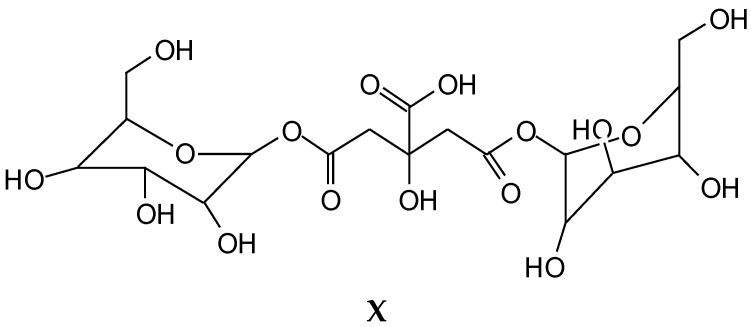
- 689 Da = glucose-citric acid-glucose-citric acid (no Na^+^), structure XI
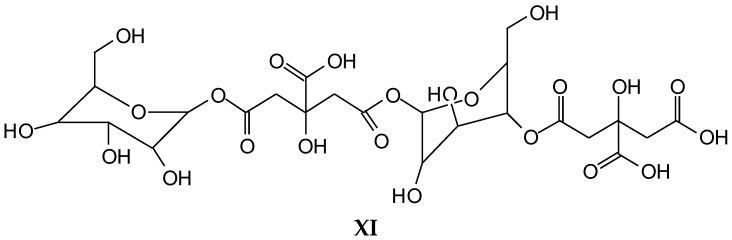
- 701 Da = citric acid-glucose (-citric acid)–citric acid (no Na^+^), structure XII
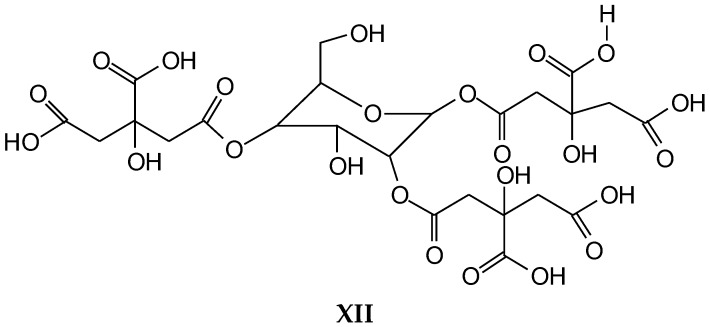

To conclude the chemical analysis, it is evident that when citric acid was used as a wood adhesive, it was able to react with both lignin and carbohydrates present in the wood.

The preparation of citric acid-bonded LVL boards appeared to be easy and practical. The citric acid solution could be properly spread onto the veneer surface, but it was observed that low density veneers showed better penetration and distribution. The same trend was observed for smoother veneers in comparison with rougher ones. The solution was applied only onto one veneer surface that then was put in contact with another veneer surface devoid of citric acid. However, the feasibility of placing face-to-face two equally treated citric acid veneers was also tested. When this latter kind of assembling was used, some severe degree of delamination was observed.

**Figure 6:**
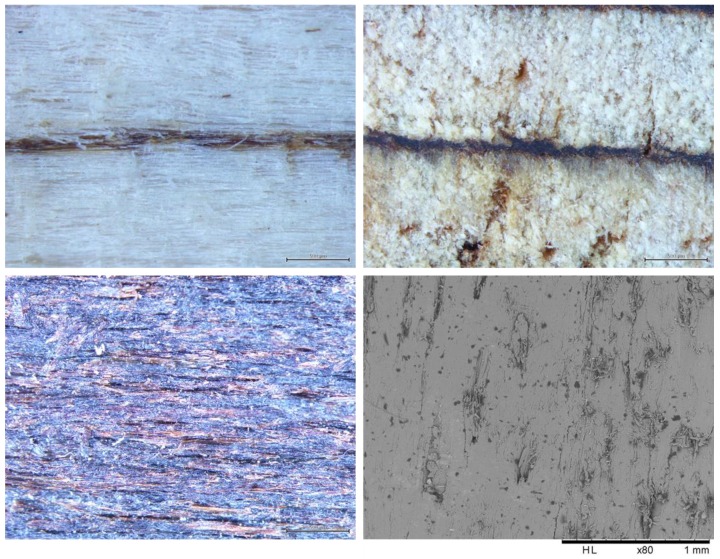
Parallel to grain (**top left**) and cross-sectional (**top right**) macroscopic view of the citric acid bond line; appearance of the inner surface of the veneer after reaction with citric acid (**down left**), and scanning electronic micrography (80×) of the glued joint (**down right**).

Figure 6 shows the macroscopic view (500 μm) of the citric acid reaction into the veneer’s surface and the thickness profile of the LVL board (top left and right). The reaction between citric acid and the wood yielded a dark-brown bond line (left). This bond line might be formed by the reaction of wood carbohydrates, lignin, and their model compounds with citric acid as can as well be caused by the oxidation induced by the citric acid and heat on the wood constituents. The image down left in Figure 6 shows the surface of the veneers after treatment with citric acid before pressing and does not show any particular alteration of the surface before heat was applied. The SEM micrography (80×) of the glued joint is also shown in Figure 6 (down right). It was not possible to identify the bond line between the veneers even when a 3000× magnification (30 μm) was used. It might well be that the veneers boundary melted and that possibly materials from the two veneers surfaces flowed into each other. A similar case has already been reported when citric acid has been used to improve wood joints bond lines during wood friction welding [5]. In this case, it was the combination of acid and temperature caused by the welding that cause constituent entanglement and interpenetration at the joint interphase [5]. It can be thought that a similar behavior can occur here although this cannot be ascertained with the present information. It is believed that the hot-pressing schedule used played an important role in this process, since it helped to keep the closest contact between the veneers. This would allow the reaction between the untreated surface and the citric acid treated surface, thus increasing the interfacial density.

**Figure 7:**
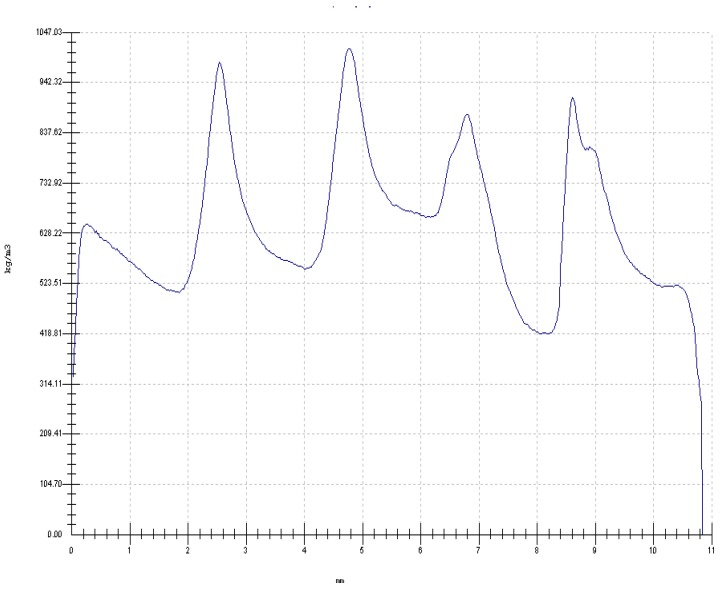
X-Ray density profile of the 5-layer citric acid-bonded LVL (ρ = 610 kg/m^3^). The x-axis details the mm thickness of the panel (from 0 to 11 mm). The y-axis details the density in kg/m^3^ (scale from 0.00 to 1047 kg/m^3^).

The X-ray densitometry of the samples (Figure 7) shows that the bond line interface presented density peaks that reached 1015 kg/m^3^, which is almost three times higher than the density of the veneers before pressing. Conversely, the regions between the veneers presented a much lower density almost comparable to that of not pressed veneers. This profile appears like that usually found on wood welded joints, where the bond line between the wood samples presents high density [11].

The properties of the citric acid bonded LVL are presented in Table 1. The low variation of the properties should be highlighted, such as coefficients of variation lower than 10%. It can be observed that the glue line shear strength exceeded the minimum required by EN-314-2 (1 N/mm^2^) [8]. Additionally, bending strength (*f*_m_) and stiffness (*E*_M_) and parallel compression strength (*f*_c,0_) were similar or higher than those found in the literature for LVL bonded with synthetic resins. All the bending samples failed in tension on the outer bottom side veneer. Kurt et al. [12] produced poplar phenol-formaldehyde (PF)-bonded LVL, and they found (*f*_c,0_) values between 46.4 and 57.98 N/mm^2^. Rahayu et al. [13] performed a comprehensive study about the utilization of new poplars cultivars to produce LVL bonded with polyvinyl acetate. In general, the bending strength (*f*_m_) ranged between 47.4 and 64.8 N/mm^2^, while the bending stiffness (*E*_M_) was from 7250 to 10,312 N/mm^2^. Wang et al. [14] found *f*_m_ ranged from 79.9 to 90.5 N/mm^2^ whereas the *E*_M_ ranged from 8362–9185 N/mm^2^ for non-reinforced poplar PF resin-bonded LVL. Recently, Bal [15] also evaluated the properties of PF-bonded poplar LVL and found 66.1 N/mm^2^ for *f*_m_ and 5433 N/mm^2^ for E_M_. The values presented here were also between the range presented by the Forest Products Laboratory [16] for *E*_M_ (4900–12,500 N/mm^2^) and *f*_m_ (33.8–88.2 N/mm^2^).

The thickness swelling was slightly higher, but the water absorption was lower than usually found in the literature for LVL [15]. Probably the main reason behind this behavior is the level of pressure applied. This pressure was chosen to allow with the same operation both consolidation and densification of the LVL board to improve its mechanical properties. The density of the LVL billet before pressing was about 478 kg/m^3^ while the density of the consolidated LVL was 607 kg/m^3^, which means a 27.1% of densification ratio. Additionally, this pressure yielded denser bond lines as seen in Figure 7. It means that certain levels of compression stresses are introduced in the assembly, and when the board gets in contact with water, these are released leading to thickness swelling above the ones usually found.

Control experiments to try to bond the same veneers but without any addition of citric acid were also carried out, but there was no bond formed, literally the plywood fell apart. There is also previous experience using this approach, where plywood veneers were pressed without anything else to form plywood [17]. The bond did indeed form, but to reach values vaguely similar to those obtained in the present experiments, only at temperatures of 250 °C for a pressing time of 75 min at high pressure [17], while in the case of the present experiments, the plywood was only pressed for 20 min at 180 °C. In the same reference, welding was recorded by scanning electron microscopy and X-ray microdensitometry showing loss of wood cells morphology in the welded interphase [17].

## 4. Conclusions

The reaction of citric acid with wood veneers showed that reactions of citric acid occurred with both lignin and carbohydrate constituents of wood. The reactions occurring are esterifications between the carboxylic acid functions of citric acid and the numerous aromatic and aliphatic hydroxyl groups of the main wood constituents. These results confirm under the conditions used for wood panel pressing the hypothesis of citric acid esterifying hydroxyl groups in the wood constituents advanced by the Umemura group for different panels [1,2], while lignin rearrangements also contributed to the improvements in water resistance as indicated by other research groups [5]. The applied results on bonding wide flat wood surfaces such as veneers to obtain LVL panels or plywood have shown that citric acid has great potential to be used as bio-binder for wood veneers.

## Figures and Tables

**Table 1:** Mechanical and physical properties of the 10%-citric acid bonded LVL.

*f*_m_ (N/mm^2^)	*E*_M_ (N/mm^2^)	*f*_c,0_ (N/mm^2^)	*f*_gv,0_ (N/mm^2^)	TS2h (%)	WA2h (%)	TS24h (%)	WA24h (%)	PTS (%)	Density (kg/m^3^)	EMC (%)
101.2 (7.45)	13,177 (403.7)	54.3 (4.54)	2.81 (0.29)	6.1 (0.65)	24.3 (2.01)	9.2 (0.84)	44.9 (2.11)	2.59 (0.77)	607 (10.9)	9.01 (0.54)

Standard deviation between parenthesis; *f*_m_: modulus of rupture; *E*_M_: modulus of elasticity; *f*_c,0_: compression parallel to the grain strength; *f*_gv,0_: glue-line shear strength; TS: thickness swelling; WA: water absorption; PTS: permanent thickness swelling; EMC: equilibrium moisture content.
